# Surgical Anatomy and Approaches of the Anterior Cranial Fossa from a Transcranial and Endonasal Perspective

**DOI:** 10.3390/cancers15092587

**Published:** 2023-05-01

**Authors:** Pedro Plou, Simona Serioli, Luciano C. P. C. Leonel, A. Yohan Alexander, Edoardo Agosti, Larissa Vilany, Stephen Graepel, Garret Choby, Carlos D. Pinheiro-Neto, Maria Peris-Celda

**Affiliations:** 1 Department of Neurologic Surgery, Mayo Clinic, Rochester, MN 55905, USA; 2 Mayo Clinic Rhoton Neurosurgery and Otolaryngology Surgical Anatomy Program, Rochesrer, MN 55905, USA; 3 Department of Neurosurgery, Hospital Italiano de Buenos Aires, Buenos Aires C1181ACH, Argentina; 4 Division of Neurosurgery, Department of Medical and Surgical Specialties, Radiological Sciences and Public Health, University of Brescia, 25121 Brescia, Italy; 5 Department of Otorhinolaryngology Head and Neck Surgery, Mayo Clinic, Rochester, MN 55905, USA

**Keywords:** anterior cranial fossa, endoscopic endonasal approach, surgical anatomy, skull base surgery, transcranial approach

## Abstract

**Simple Summary:**

The technical development in skull base surgery, especially to the anterior cranial fossa, has allowed for improvement in the quality of life and the long-term prognosis of patients suffering from different types of tumors that can affect this anatomical area. Applying these advanced techniques requires a thorough understanding of the skull base anatomy and surrounding structures to perform surgery without potential intraoperative complications related to the pathology itself as well as potential complications related to the surgical approach that has been chosen to treat this pathology. The purpose of this paper is to offer a comprehensive review of the surgical anatomy of the anterior skull base and the technical nuances of the surgical approaches that are more frequently used in this region. Detailed knowledge of these aspects is essential both for the choice of the surgical approach and for its correct execution when an anterior cranial fossa lesion need to be resected.

**Abstract:**

The anterior cranial fossa (ACF) is a complex anatomical region that can be affected by a broad spectrum of pathology. For the surgical treatment of these lesions, many approaches have been described, each of them with different scope and potential surgical complications, often associated with significant morbidity. Traditionally, tumors involving the ACF have been operated by transcranial approaches; however, in the last two decades, endoscopic endonasal approaches (EEAs) have been gaining popularity. In this work, the authors review and describe the anatomical aspects of the ACF and the technical nuances of transcranial and endoscopic approaches for tumors located in this region. Four approaches were performed in embalmed cadaveric specimens and the key steps were documented. Four illustrative cases of ACF tumors were selected to demonstrate the clinical application of anatomical and technical knowledge, which are essential in the preoperative decision-making process.

## 1. Introduction

The anterior cranial fossa (ACF) constitutes the most anterior part of the endocranial surface of the skull base and accommodates the basal cortex of the frontal lobes and the olfactory bulbs. It is anteriorly limited by the orbital rim, while posteriorly, the border with the middle cranial fossa is represented by the sphenoid ridge and the sphenoidal limbus, including the posterior limit of the optic canal. Extracranially, the anterior skull base is inferiorly related to the sphenoid sinus, the nasal cavity, and the orbit [1]. 

This anatomical region can be affected by a broad spectrum of neoplastic and infectious pathologies, which can extend both intracranially and extracranially. Most of the malignant tumors arise from the nasal cavity, paranasal sinus, orbit, salivary glands, or metastatic lesions [2,3].

Of lesions involving the ACF, meningiomas represent approximately 12–20% [4], hemangiopericytomas (now known as solitary fibrous tumors) are rarely found, especially in the nose and paranasal cavity [5]. Esthesioneuroblastoma, also known as olfactory neuroblastoma, represents 3% of intracranial tumors. This tumor usually arises from the olfactory epithelium, involving the lamina cribrosa of the ethmoid bone and the medial aspect of the ACF [6].

Lesions that involve the ACF can be approached either from above, through transcranial approaches, or from below, through endoscopic endonasal approaches (EEAs). Traditional transcranial approaches include pterional, frontal, bifrontal, and transbasal approaches with their variants [7]. More recently, the supraorbital approach has gained popularity as a less invasive approach to the anterior and medial cranial fossa [8,9].

The concept of the endonasal approach was conceived more than a century ago [10], initially limited to the pituitary fossa. First, the introduction of the microscope—and more recently, the endoscope—improved the accuracy of the approach with better visualization and illumination [11,12,13]. The anatomical conformation of the medial part of the ACF, formed by the nasal cavity and sphenoidal sinus, made these structures suitable for an endoscopic approach to access and treat skull base pathology. In the last two decades, the EEA has gained growing importance, especially as reconstructive techniques, biotechnology, and anatomical knowledge have advanced to allow safe and effective access to pathologies from the anterior cranial fossa to the odontoid process [14,15,16].

Each approach has its own scope as well as its rate of complications. Therefore, in the decision-making process, this aspect must be taken into consideration in conjunction with the patient’s anatomy and the nature of the pathology. The aim of this work is to review the anatomical nuances of the ACF and the different approaches for tumors located in this region. 

## 2. Method 

All aspects of this study were approved by the Institutional Review Board and Biospecimens Committee (17–005898), as required by standard protocols. 

We reviewed the anatomical aspect and technical nuances of the approaches to the ACF.

Four approaches to the ACF were performed on six embalmed and latex-injected cadaveric heads. Four of them were used for the transcranial approaches, while two were used for endoscopic dissection. The key steps of each approach were illustrated.

For illustrative purposes, four cases of ACF tumors operated by the senior authors (MPC and CPN) were selected.

## 3. Results 

### 3.1. Anterior Cranial Fossa Anatomy 

**Figure 1:**
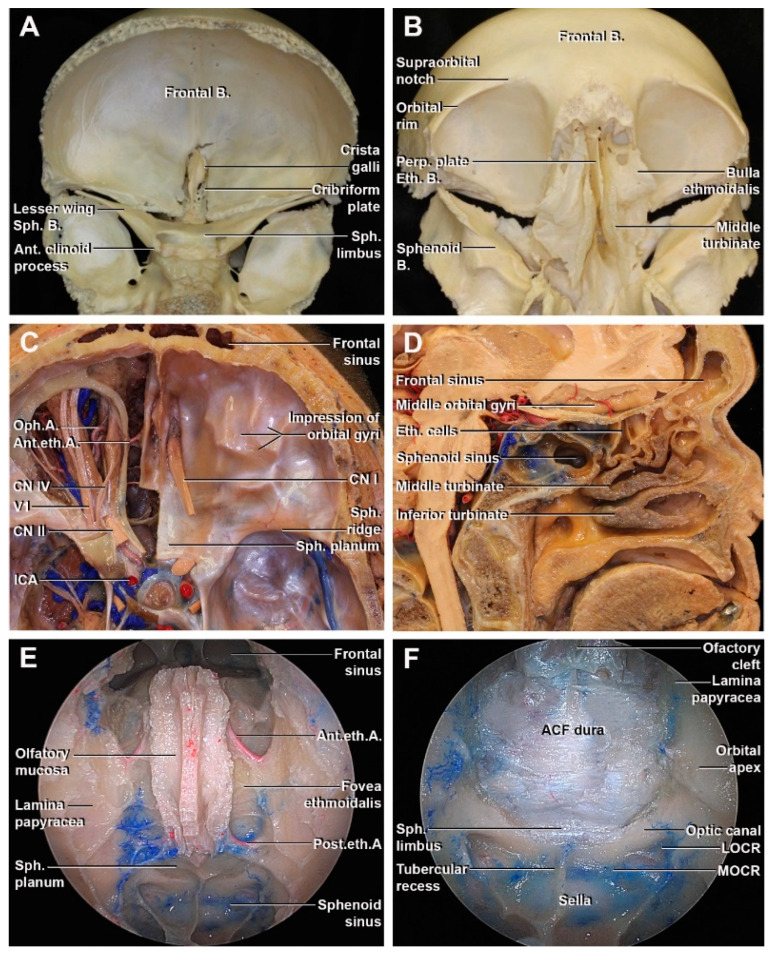
Anterior cranial fossa anatomy. (**A**,**B**) Intracranial and extracranial view of the bony anatomy. (**C**) Anatomic dissection of the ACF showing the relationship between the neurovascular structures. (**D**) Sagittal section of an anatomic dissection showing the relation of the nasal and paranasal structures with the ACF. (**E**,**F**) Endoscopic view of the frontal sinus, nasal cavity roof, sphenoidal sinus, and (**E**) ACF dura and sphenoidal limbus (**F**). A: artery; ACA: anterior cerebral artery; ACF: anterior cranial fossa; Ant.: anterior; B: bone; CN: cranial nerve; Eth: ethmoidal; ICA: internal carotid artery; LOCR: lateral optic-carotid recess; MOCR: medial optic-carotid recess Oph: ophthalmic; Perp: perpendicular; Post: posterior; Sph: sphenoidal.

The anterior cranial fossa occupies the most anterior aspect of the skull base, and it is laterally delimited from the middle cranial fossa by the sphenoidal ridge and medially by the sphenoidal limbus (Figure 1A,C). The lateral part of the ACF is formed by the orbital plates of the frontal bone that composes the anterior two-thirds and the lesser wing of the sphenoid bone posteriorly. In the midline, the frontal bone harbors the ethmoidal notch, a quadrangular space laterally limited by the orbital plates, where the ethmoid bone articulates. The ethmoid bone is characterized by a cuboidal-shaped and consists of a horizontal cribriform plate with the crista galli, a perpendicular plate in the midline, and laterally, the labyrinth with the ethmoid air cells (Figure 1B). The cribriform plate constitutes the median part of the anterior cranial fossa floor and the roof of the nasal cavity. It is characterized by numerous foramina through which the nerve branches of the olfactory nerve pass. The crista galli, a triangular process that arises from the most anterior part of the cribriform plate, serves as an insertion to the falx in the midline. From the extracranial view, the ethmoid bone forms the roof of the nasal cavity, part of the paranasal sinuses, and part of the medial wall of the orbit. In the midline, the perpendicular plate articulates with the vomer and the quadrangular cartilage to form the nasal septum. The ethmoid labyrinth constitutes part of the lateral nasal cavity wall and is anteriorly related to the frontal sinus and posteriorly to the sphenoid sinus. The lamina papyracea, the most lateral surface of the ethmoid cells is a thin bony layer that constitutes part of the medial wall of the orbit (Figure 1E). The anterior and posterior ethmoidal arteries run from lateral to medial through the most superior aspect of the ethmoid labyrinth. These arteries arise in the orbit from the ophthalmic artery and pass through the anterior and posterior ethmoidal canals in the roof of the ethmoid labyrinth. They intracranially send branches to the meninges and falx cerebri, and extracranially to the septum and lateral wall of the nose. The posterior ethmoidal artery has a smaller caliber and runs just anterior to the planum sphenoidale. The anterior ethmoidal artery, which represents a landmark for the fovea ethmoidalis, is located just posterior to the nasofrontal recess [17].

The posterior part of the ACF is formed by the sphenoid bone. The sphenoidal crest, a middle protrusion of the sphenoidal conchae, articulates with the perpendicular plate of ethmoids. The sphenoid sinus is located in the body of the sphenoid, superiorly delimited by the planum sphenoidale, floor of the ACF (Figure 1D,F). The sphenoid ostium, usually identified medial to the superior turbinate, connects the sphenoidal cavity with the sphenoethmoidal recess. The lesser wing of the sphenoid articulates with the frontal bone to complete the posterior one-third of the lateral part of the ACF.

### 3.2. Transcranial Approaches to the Anterior Cranial Fossa 

#### 3.2.1. Bifrontal/Transbasal Approach

**Figure 2:**
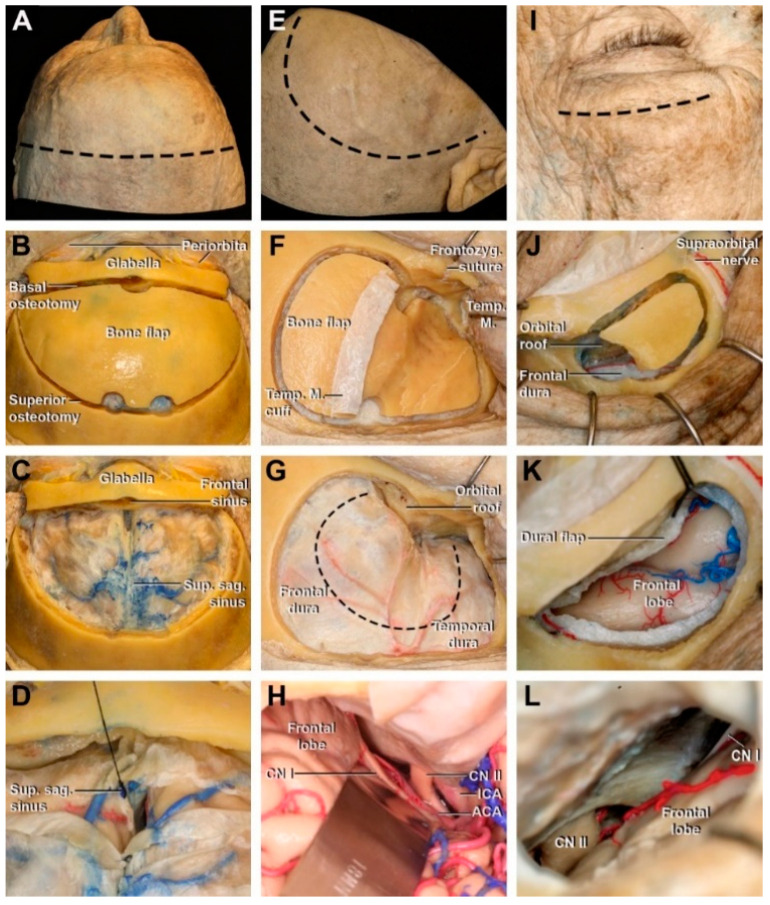
Transcranial approaches to the anterior cranial fossa performed in cadaveric specimens. (**A**–**D**) Bifrontal transbasal approach. (**A**) Supine positioning with the head deflected and a bicoronal incision from one zygomatic root to the other (black dash line). (**B**) Bifrontal craniotomy above the orbital rim and the glabella. (**C**) Frontal dural exposure after craniotomy. (**D**) Dural opening and division of the inferior aspect of the superior sagittal sinus. (**E**–**H**) Pterional approach. (**E**) Skin incision from the midline to the zygomatic root posterior to the hair line (black dash line). (**F**) Frontotemporosphenoidal craniotomy with two burr-holes. A temporal muscle cuff is left in the bone flap to reattach the muscle after the bone is replaced. (**G**) Frontal and temporal dura exposure. The orbital roof is drilled and flattened. Dash line indicates the “C” shape dura incision. (**H**) Subfrontal corridor exposure. (**I**) Skin incision in the superior aspect of the eyebrow (black dash line). (**J**) The bur-hole is inferior to the temporal line and the craniotomy extends up to the supraorbital notch. (**K**) The dural flap inferiorly retracted, exposing the frontal lobe. (**L**) View of the subfrontal corridor after brain relaxation. ACA: anterior cerebral artery; CN: cranial nerve; Frontozig: frontozygomatic; ICA: internal carotid artery; Orbitofr.: orbitofrontal; Sup. sag: superior sagittal. Temp. M: temporal muscle.

The first concept of the bifrontal approach was described by Frazier in 1913 [18]. Since then, several modifications and extensions have been proposed. A bicoronal skin incision extending from the level of one zygomatic root to the contralateral zygomatic root is performed posterior to the hairline, followed by wide scalp flap elevation (Figure 2A). The pericranium should be dissected and preserved in case a paricranial flap is required. The anterior part of the frontal bone is bilaterally exposed. Two burr holes are placed superior to the Mc Carty´s point and one or two at the midline, just lateral to the superior sagittal sinus as much posterior as the craniotomy needs to be. Then, a basal osteotomy superior to the orbital rim connects the two lateral burr holes. Two superior osteotomies connect the parasagittal burr hole with each lateral one (Figure 2B). For the transbasal extension, another osteotomy is placed over the frontonasal suture and extends laterally to the level of the supraorbital notch. Once the dura is exposed, the superior sagittal sinus can be sectioned in the anterior portion (Figure 2C,D). This extension allows more extradural exposure of the anterior cranial base and opens to the nasal cavity [19].

#### 3.2.2. Pterional Approach 

The pterional approach, described by Yasargil in 1975, is one of the most frequently used approaches in neurosurgery [20]. It involves a fronto-temporo-sphenoidal craniotomy behind the orbital rim, centered in the pterion. The skin incision starts at the midline, posterior to the hairline and ends at the level of the zygomatic root; however, it can be carried all the way to the contralateral midpupillary line depending on how much is needed to extend the craniotomy to the midline (Figure 2E). Once the scalp is elevated, an interfacial, subfascial, or one-layer dissections of the temporal muscle avoid injury to the frontal branch of the facial nerve. The first burr hole is placed below the temporal line and just posterior to the zygomatic process of the frontal bone. One to three burr holes can be performed in the frontal or temporal bone. More burr holes are useful in the elderly where the dura is finer and more adherent to the bone. The osteotomies connecting the burr holes involve the frontal, temporal, and greater wing of the sphenoid bone (Figure 2F). The craniotomy is completed by drilling out the most lateral part of the sphenoid wing and the orbital roof to flatten the floor of the ACF (Figure 2G). For the ACF, the subfrontal space is the main surgical corridor (Figure 2H), while splitting the sylvian fissure opens the corridor posterior to the sphenoid ridge [20,21]. 

#### 3.2.3. Eyebrow Supraorbital Approach 

The supraorbital eyebrow approach was first described in 1998 to treat anterior circulation aneurysms and was later popularized for lesions of the ACF [8,9]. This approach involves an eyebrow incision lateral to the supraorbital notch (Figure 2I). The scalp flap is superiorly retracted, preserving the supraorbital nerve, and the temporal muscle is posteriorly dissected 1.5 cm. The burr hole is placed inferior to the temporal line and posterior to the zygomatic process of the frontal bone. The craniotomy is performed above the orbital rim, and the roof of the orbit can be flattened with the drill to favor a more basal exposure (Figure 2J). The dura is opened in a “C” shape and inferiorly retracted to expose the frontal pole and the frontal base (Figure 2I,K) [22]. 

### 3.3. Endonasal Endoscopic Approach 

EEAs to the anterior fossa require different degrees of sinonasal resection depending on the location and the extension of the pathology. When the lesion is located in the posterior aspect of the ACF (planum sphenoidale), a transplanum approach is necessary. For this EEA, an opening of the sphenoidal rostrum is necessary with an ostetomy that encompasses the planum sphenoidale. The middle and inferior turbinate are preserved as well as anterior ethmoidal cells. The superior turbinate and posterior ethmoidal cells may be resected to allow better access to the roof of the sphenoid sinus. 

If the tumor involves the anterior two-thirds of the ACF, a transcribriform approach is necessary. The classical transcribriform approach required complete removal of the ethmoidal cells, the middle and superior turbinates, frontal sinusotomy Draf III, sphenoidotomies, and resection of the superior nasal septum. Reconstruction typically requires a nasoseptal flap harvest [23,24,25,26,27].

**Figure 3:**
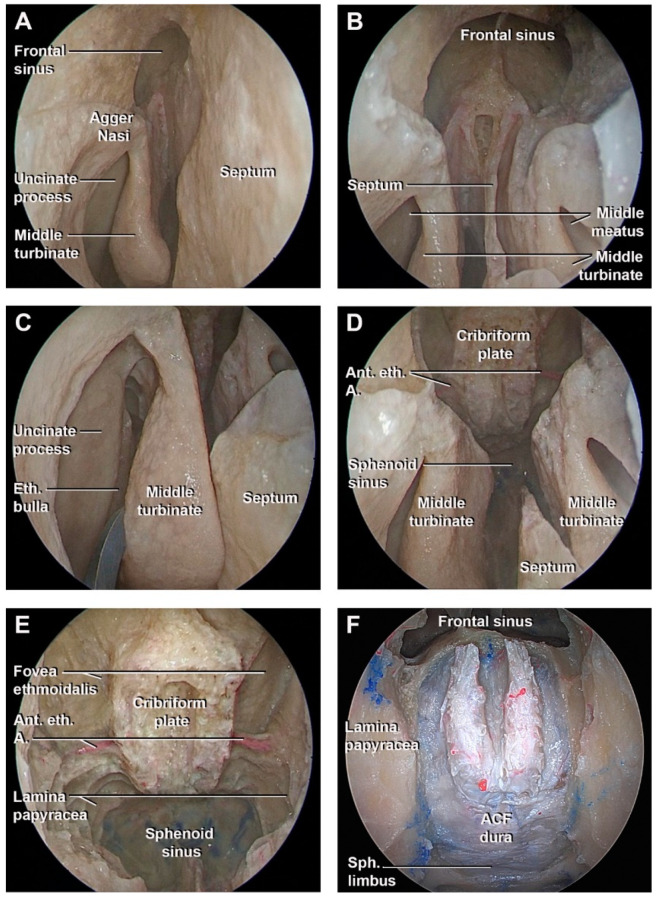
Endoscopic superior ethmoidal approach performed in cadaveric specimens. (**A**) Exposure of the agger nasi region and frontal sinus ostium. (**B**) Frontal sinus Draf III and superior septostomy exposing the posterior wall of the frontal sinus. (**C**) Middle meatus structures preserved. (**D**) Superior part of the ethmoid labyrinth resected and cribriform plate exposure. (**E**) Exposure of the anterior cranial fossa floor. (**F**) Dural exposure after bony removal. A: artery; ACF: anterior cranial fossa; Ag.N: ager nasi; Ant.: anterior; Eth: ethmoidal; Sph: sphenoidal.

Recently, a more conservative approach for nasal structures has been described by Peris-Celda et al. [28]. This technique avoids the resection of the middle turbinate, uncinate process, and bulla ethmoidalis if they are not involved in the pathology. After nasoseptal flap harvest, a Draf III frontal sinusotomy is performed, preserving the anterior attachment of the middle turbinate. A limited superior septectomy is accomplished just inferior to the frontal sinus floor followed by the drilling of the frontal sinus floor to complete Draf III (Figure 3A,B). The anterior limit of the olfactory cleft is exposed. The anterior septectomy is posteriorly enlarged no more than 1 cm below the cribriform plate. Both middle turbinates are lateralized and the superior turbinate is resected. The vertical attachment of the middle turbinate is posteriorly respected, preserving the anterior attachment to the lateral wall of the nose (Figure 3C,D). This provides access to the superior compartment of the ethmoid bone. The ethmoidectomy is completed by resecting the superior portion of the basal lamella and the superior aspect of the anterior and posterior ethmoid cells, exposing the lamina papyracea. The sphenoid rostrum is bilaterally opened to expose the planum sphenoidale. Once the anterior fossa floor is exposed, four osteotomies are performed, two medial to the lamina papyracea, one just posterior to the posterior wall of the frontal sinus, and the last one at the level of the sphenoid rostrum (Figure 3E). The bone between the osteotomies can be drilled, followed by resection of the paper-thin bone to expose the dura mater (Figure 3F) [27,28].

**Figure 4:**
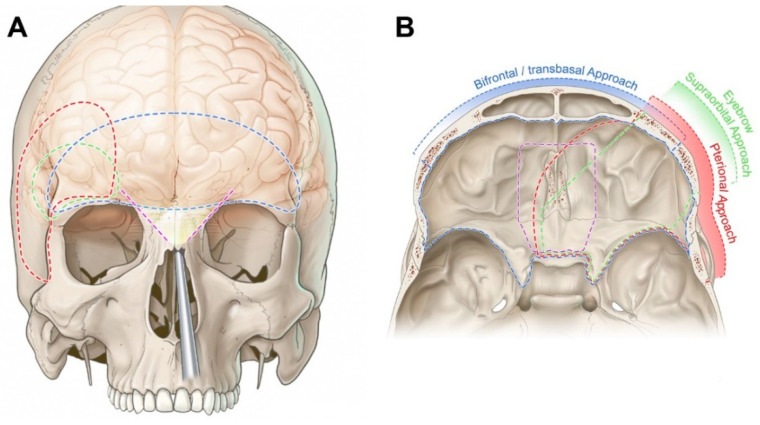
Illustration of the anterior cranial fossa approaches. (**A**) Craniotomy area is represented by the dashed lines, anterior view. (**B**) area of exposure of each approach is illustrated, superior view. Red: pterional; Blue: bifrontal; Green: supraorbital; Purple represents the angle of view of the endonasal endoscopic approach (**A**) and the area of exposure (**B**).

Figure 4 illustrates and summarizes the craniotomies (Figure 4A) and the surgical corridors (Figure 4B) of the approaches described above. 

### 3.4. Illustrative Cases 

#### 3.4.1. Case 1

**Figure 5:**
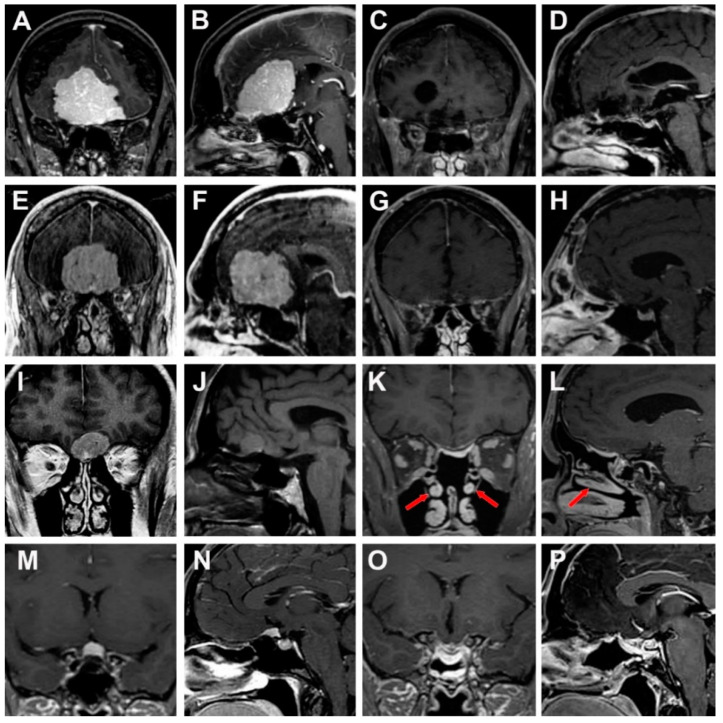
Preoperative and postoperative magnetic resonance imagines (MRI) of the clinical cases. (**A**–**D**) Case 1, preoperative coronal and sagittal view showing an anterior skull base meningioma centered over the planum sphenoidale (**A**,**B**). Postoperative coronal and sagittal view confirming gross total resection (**C**,**D**). (**E**–**H**) Case 2, preoperative coronal and sagittal section of olfactory groove meningioma (**E**,**F**) and postoperative coronal and sagittal view documented the gross total resection of the lesion (**G**,**H**) (**I**–**L**) Case 3, preoperative coronal and sagittal view of olfactory groove meningioma (**I**,**J**). Postoperative MRI after endoscopic transnasal superior ethmoidal approach with preservation of the middle turbinate (red arrow) (**K**,**L**). (**M**–**P**) Case 4, preoperative coronal and sagittal section of tuberculum sellae meningioma (**M**,**N**) and postoperative MRI after endoscopic endonasal transplanum approach (**O**,**P**).

A 49-year-old man with a history of developmental disabilities and psychiatric disorders presented with memory impairment and personality changes. Brain MRI revealed a large anterior skull base meningioma (5.7 cm × 5.1 cm × 4.7 cm) centered over the planum sphenoidale with vasogenic edema. The lesion caused an important mass effect with deviation of anterior cerebral arteries and obliteration of the anterior horns of lateral ventricles (Figure 5A,B). The patient underwent a frontotemporal approach, with orbital osteotomy, and gross total resection of the tumor was achieved. The patient recovered well from the operation without new neurological deficits. The histopathologic examination revealed a WHO II grade meningioma, and WHO I grade meningioma for the small adjacent tumor. The follow-up MRI documented the gross total resection of the lesion, without signs of recurrence or remnants (Figure 5C,D). 

#### 3.4.2. Case 2 

A 68-year-old male presented an episode of unconsciousness, associated with personality changes in recent years, including abulia, slow gait, and unsteadiness. He also reported loss of smell and taste. A brain MRI documented a large olfactory groove meningioma (5.6 cm × 5.5 cm × 4.4 cm) with bilateral frontal vasogenic edema and bilateral compression of the frontal horns of lateral ventricles (Figure 5E,F). The patient underwent a bifrontal craniotomy with cranialization of the frontal sinus, with abdominal fat graft and pericranial flap. There were no postoperative complications. The final histopathologic examination confirmed a WHO II grade meningioma, characterized by high cellularity and parenchymal invasion. Gross total resection was achieved (Figure 5G,H). 

#### 3.4.3. Case 3

**Figure 6:**
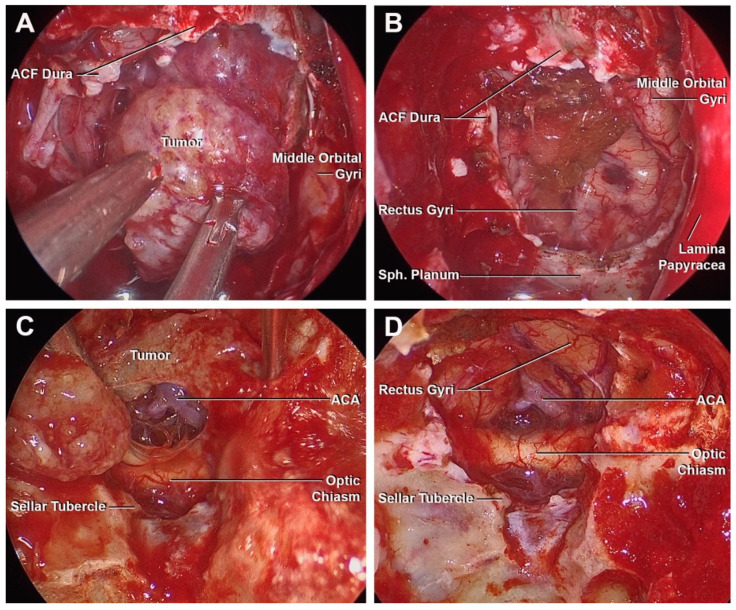
Endoscopic intraoperative images of case 3 (**A**,**B**) and case 4 (**C**,**D**). (**A**) Dura opened and tumor exposure through the endoscopic superior ethmoidal approach for an olfactory grove meningioma. (**B**) Basal surface of the frontal lobe was exposed after the tumor resection. (**C**) Tumor exposure through endoscopic transplanum approach. (**D**) Basal surface of the frontal lobe and suprachiasmatic surface exposure after tumor resection.

A 56-year-old male experienced an inability to smell and persistent difficulty in right hand and arm movements for over a year. Brain MRI in the workup for right upper limb weakness revealed an olfactory groove meningioma (2.6 cm × 2.0 cm × 2.4 cm), characterized by foci of calcification in the left central portion of the tumor (Figure 5I,J). The arm weakness was deemed unrelated to the tumor. The patient underwent an endoscopic endonasal superior ethmoidal approach with preservation of the middle turbinates and reconstruction of the skull base defect with an inlay fascia lata graft and nasoseptal flap (Figure 6A,B). The postoperative course was uneventful. Pathology was reported as meningothelial meningioma (WHO grade I). The postoperative MRI evidenced no residual tumor (Figure 5I,K). 

#### 3.4.4. Case 4 

A 35-year-old woman underwent a brain MRI scan for evaluation of headaches. During the diagnostic investigation, incidental meningioma was found located in the planum sphenoidale (Figure 5M,N). The tumor was initially observed and demonstrated growth over an 11-month time frame. The patient did not develop any neurological deficits with normal visual fields. She underwent an endoscopic endonasal transplanum transtuberculum approach, followed by a left-sided nasoseptal flap without postoperative complications (Figure 6C,D). The histological analysis was consistent with WHO I grade meningioma. Postoperative MRI evidenced no residual or recurrent tumor (Figure 5O,P). 

## 4. Discussion

The ACF anatomy with its complex neurovascular architecture represents a challenge and requires an accurate planning to tailor the approach to the pathology [29].

Surgical techniques have greatly evolved since the first resection of an anterior skull base meningioma in 1938 [14,30]. The introduction of the microscope in the neurosurgical theater allowed a technical improvement in the dissection and resections of the tumors [11,12]. The bifrontal approach was the first approach used for ACF lesions. It favors a wide surgical exposure allowing a subfrontal and interhemispheric corridor. Unlike other transcranial approaches, the bifrontal approach can access intranasal tumor if indicated by drilling the cribriform plate. Moreover, it is possible to obtain a large pericranial flap in case skull base reconstruction is required [31]. One of the disadvantages of this technique is the opening of the frontal sinus, which carries a 5 to 14% CSF leak risk. Another consideration of this approach is the brain retraction that is needed to reach the anterior fossa floor, with the associated risk of brain retraction injury. This can become a concern due to the brain edema associated with some lesions, specifically large meningiomas [32,33,34]. The subcranial or transbasal extension of this approach, although more technically demanding and time-consuming, minimizes brain retraction and favors a more basal exposure of the ACF [35,36]. Another unfavorable aspect of the bifrontal approach, as well as its modifications, is the late visualization of the anterior cerebral artery complex and the optic apparatus [33,34,36]. This is an important point to take into account if the tumor involves a major artery or the optic nerve, as many meningiomas do in this location. 

The pterional craniotomy is one of the most common approaches used by neurosurgeons. The subfrontal corridor afforded by a pterional approach allows access to the anterior fossa. The main advantage of this approach is the early brain relaxation that can be reached by opening the cisterns and splitting the sylvian fissure. It is also important the early vascular control of the internal carotid artery and anterior cerebral arteries, often involved by ACF tumors [20,21]. For lesions that grow in the midline and rise into the interhemispheric fissure, the pterional approach does not allow a good angle of resection for the upper part. Another disadvantage, common to other transcranial approaches, is the difficulty to visualize the medial side of the ipsilateral optic nerve. However, the pterional approach allows an extradural or intradural clinoidectomy that can help decompress and better visualize the optic nerve and the optic canal [37,38]. 

The eyebrow supraorbital approach is considered a less invasive technique to reach lesions of the anterior cranial fossa or even posterior [8,9]. This access avoids brain retraction injury due to the basal exposure of the ACF. The lateral extension of this approach permits a more lateral surgical angle, allowing the visualization of the ICA and ACA complex [39]. The main disadvantage of this technique is the narrow corridor and the long distance to the contralateral side. Other cons are the risk of CSF leak due to the frontal sinus opening and scalp anesthesia, caused by damage to the supraorbital and supratrochlear nerves.

The EEAs’ popularity has dramatically increased in the last two decades. The collaboration between neurosurgeons and ENT has contributed to the development of reconstructive techniques and instruments as well as improved anatomic knowledge through an endoscopic perspective and more robust surgical training [14,40,41,42,43]. Thus, indication of the EEAs to the skull base have increased. For anterior cranial fossa tumors, the EEA allows access to the medial region posterior to the frontal sinus. Anteriorly the transcribriform-transethmoidal approach is laterally limited by the orbit and posteriorly it is limited by the orbital apex. The lamina papyracea can be removed and the orbital contents can be gently pushed laterally a few millimeters for a more lateral extension. For posterior lesions or tumors that extend to the middle cranial fossa, the transplanum approach provides access to the suprachiasmatic cistern, with the lateral boundary represented by the optic nerves. The EEA favors early devascularization of meningiomas, avoids brain retraction, and reduces hospitalization time and manipulation of neurovascular structures [14,44,45]. The main concern of the EEAs for skull base tumors is the risk of CSF leak. Even though it has been dramatically reduced since the introduction of the pedicled nasoseptal flap for endoscopic skull base reconstruction [40], the risk of CSF leak is reported between 5.4–5.56% [46,47]. More recent studies report 1.6–2.4% of CSF leak rate using a more tailored reconstruction based on the grade of the leak. These include different combinations of autologous fat and bone, synthetic materials, pedicled nasoseptal flap, and other pedicled mucosal flaps, depending on the leak degree [48,49]. The violation of the nasal structures affects the nasal airflow physiology and quality of life of the patient [50,51]. Less invasive techniques, such as the superior ethmoidectomy, preserving the middle turbinate, the bulla ethmoidalis, and the uncinate process, contribute to preserving the anatomy and physiology of the nose [28]. Another very important aspect of the EEA is the difficulty in controlling vascular structures when major arteries are significantly involved [52].

Besides anatomical considerations, the nature of the pathology must be also considered to choose the approach. Most of the malignant tumors of the ACF arise from the nasal cavity, paranasal sinus, orbit, and salivary glands [2]. The surgical management of these tumors often requires extensive resections with combined endoscopic and transcranial approaches followed by complex skull base reconstructions with pedicled flaps or even microsurgical free flaps in many cases [53]. Brain infiltration in the case of higher grade meningioma [54] or vascular encasement is relevant to choosing an approach that allows a better visualization of the tumor-brain interface. In this regard, the transbasal bifrontal approach or the EEA allows the best angle of vision from the inferior to superior direction, although safe vascular dissection in case of encasement may be challenging in EEA. Tumor vascularity is another important consideration. Hemangiopericytomas are highly vascularized lesions as well as some types of meningiomas. The main vascularization of meningiomas comes from the meningeal arteries and, for that reason, coagulation of the meningeal origin at the beginning of the procedure allows early devascularization of the tumor [55]. The EEA and transbasal bifrontal approach provide early extradural access to the vascular supply of the lesion which is often the ethmoidal arteries in the case of ACF meningiomas [14].

## 5. Conclusions

The anterior cranial fossa is a complex anatomical region that is in close relation with important neurovascular structures and is extracranially surrounded by intricate anatomy. The management of ACF tumors still represents a challenge despite the continuous technological development and less invasive neurosurgical techniques. Understanding the anatomy and technical aspects of the approaches is essential to surgical planning, decreasing the risk of complications and patient’s morbidity.
